# First Detection and Genomic Characterization of Bovine Norovirus from Yak

**DOI:** 10.3390/pathogens11020192

**Published:** 2022-01-31

**Authors:** Yuchen Cui, Xi Chen, Hua Yue, Cheng Tang

**Affiliations:** College of Animal and Veterinary Sciences, Southwest Minzu University, Chengdu 610041, China; cuiyuchen199654@sina.com (Y.C.); cx15591859198@163.com (X.C.)

**Keywords:** yak, bovine norovirus, detection, GIII.P2_GIII.4 genome

## Abstract

Yak are a unique free-grazing bovine species in high-altitude areas. The objective of this study was to investigate the presence and molecular characteristics of BNoV in yak. A total of 205 diarrheal samples of yak (aged ≤ 3 months) were collected from 10 farms in Sichuan Province, China, from May 2018 to October 2020, and four samples were detected as BNoV-positive with RT-PCR. Moreover, a nearly full-length genome of SMU-YAK-J1 containing three complete ORFs was successfully sequenced. Sequence analysis with only nine genome sequences of the GIII genogroup showed that SMU-YAK-J1 was most closely related with GIII.P2 GIII.4, sharing 90.9% gnomic nucleotide identity, but only shared 71.6–85.9% with other genotypes, which confirmed that SMU-YAK-J1 belongs to genotype GIII.P2 GIII.4. However, compared with the sole genome of GIII.4 in GenBank, the BNoV in this study also exhibited many unique amino acid changes among all the three ORFs, which may represent the unique genetic evolution of BNoV in yak. This study first determined the presence of BNoV in yak, contributing to a better understanding of the prevalence and genetic evolution of BNoV.

## 1. Introduction

Noroviruses (NoV) are important pathogens that cause gastroenteritis in children and young animals [1,2,3]. They can be classified into ten genogroups (GI–GX) based on the complete VP1 protein [4]. Since VP1 and RdRp of NoV can evolve independently, it is not entirely accurate to describe the VP1 protein alone. Consequently, a dual nomenclature system has been proposed, considering the phylogenetic relationships of both partial RdRp (P) and capsid-coding sequences; this system is now used routinely in the molecular typing of human NoV [5,6].

Bovine NoV (BNoV) is classified into genogroup III (GIII) and can be further divided into four distinct genotypes: GIII.1, GIII.2, GIII.3 and GIII.4 based on complete VP1 [5]. GIII.1 appears to be more virulent than GIII.2, whereas GIII.2 is the most prevalent [3,7]. According to the dual nomenclature system, BNoV GIII could be reclassified as genotypes GIII.P1_GIII.1, GIII.P2_GIII.2, GIII.P1_GIII.2, GIII.P2_GIII.1 [4] and GIII.P2_GIII.4 [8]. Currently, GIII.P1_GIII.1 has been detected in nine countries; GIII.P2_GIII.2 is the most prevalent worldwide and was detected in 17 countries [9,10,11,12,13,14,15,16,17,18,19,20,21,22,23]. In 2019, a BNoV identified in a dairy cow in China was proposed to be GIII.P2_GIII.4 [8]. Since the complete VP1 and RdRp sequences of BNoV in GenBank are relatively limited, more sequences need to be identified to further refine the dual nomenclature system of BNoV.

Currently, only nine complete genome sequences of BNoV are available in GenBank: one from GIII.P1_GIII.1, seven from GIII.P2_GIII.2 and one from GIII.P2_GIII.4. Analysis of all the nine complete NoV genomes available in the GenBank database showed that these linear RNA genomes (7311–7342 bp in size) were organized into three open reading frames (ORFs). ORF1 is 1685 amino acids (aa) in length, starting from the 5′ end of the genome, and encodes viral nonstructural proteins, including the P48, NTPase, P22, VPg, pro and RdRp proteins. RdRp is a key enzyme responsible for transcription, replication of the viral genome and accurate initiation of RNA synthesis, which is essential for preventing the loss of viral genetic information [24,25,26]. In addition, the RdRp gene is usually targeted by RT-PCR because it contains highly conserved regions. ORF2 codes for the 525-aa VP1 protein, which is a significant structural component of NoVs and plays a role in viral binding to ABO histo-blood group antigens (HBGAs), receptor recognition, host specificity and immunogenicity [24]. Recently, several recombination events have been detected at the ORF1/ORF2 junction, thus generating different genotypes. ORF3 encodes the minor capsid protein (VP2), which is composed of 224 aa; VP2 is a hypervariable region in NoVs and may function in maintaining the stability of NoV particles [27].

Yak (*Bos grunniens*) belong to the genus *Bos* within the family Bovidae and is a unique long-haired bovine species inhabiting the Qinghai–Tibet Plateau [28]. There are over 14 million yak in the world; they are distributed in the high-altitude regions of China, India, Nepal, Pakistan, Kyrgyzstan, Mongolia, and the Russian Federation, and are mainly found in the Qinghai–Tibet Plateau (above 3000–5000 m) in China. Yak are essential animals that support the livelihood of the local Tibetan people, providing meat, milk, leather, and fuel (feces) and serving as a mode of transport [29]. Diarrhea is a common disease in yak, which causes serious economic losses; thus far, bovine viral diarrhea virus, bovine coronavirus and a group of bovine rotaviruses and neboviruses have been identified as common diarrhea-causing viruses in yak [30,31,32,33]. However, there is no information regarding norovirus infection in yak. Our previous studies confirmed that BNoV circulates widely in dairy cows in China [23]; accordingly, the objective of the present study was to investigate the presence and molecular characteristics of BNoV in yak.

## 2. Results

### 2.1. BNoV Detection

Of the 205 diarrhea samples analyzed, four (1.95%) were detected as BNoV-positive samples using RT-PCR and four RdRp fragments were obtained. Positive samples were found at four out of the ten farms studied. BLAST alignments showed that the four sequences had highest homology of 88.0–96.6% nt identity (82.9–96.2% aa identity) with GIII.P2_GIII.2, indicating that BNoV is emerging in yak in China.

### 2.2. Genomic Characterization of SMU-YAK-J1 in Yak

**Figure 1:**
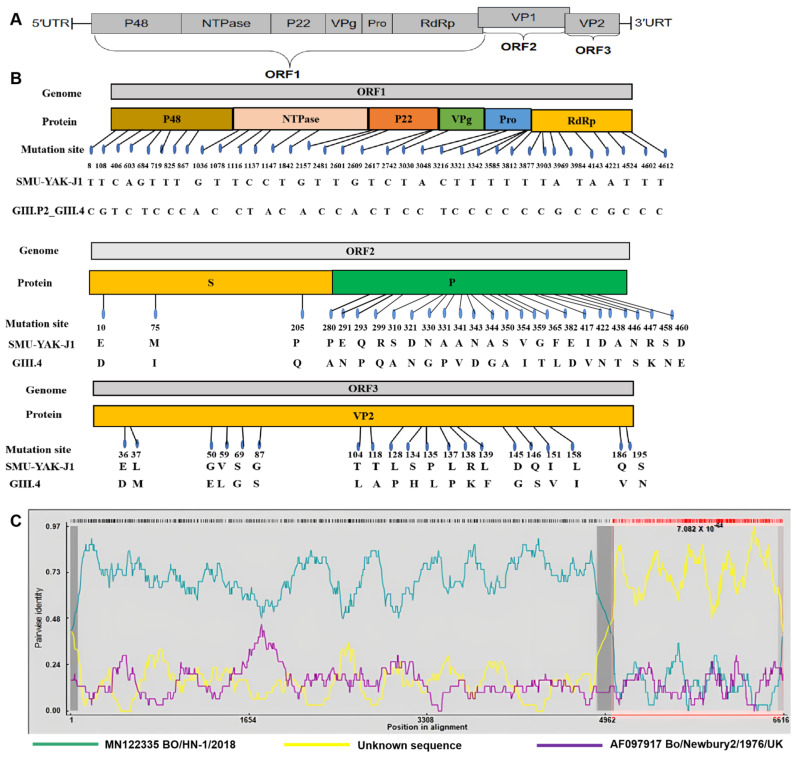
(**A**) Genomic organization of the BNoV from this study. (**B**) The aa differences between SMU-YAK-J1 and GIII.P2_GIII.4 in ORF1, ORF2 and ORF3. (**C**) Recombination analysis of RDP 4.0 supported by RDP, Chimaera, BootScan, 3Seq, GeneConv, MaxChi and SiScan, analysis of the ORF1/ORF2 region. The recombination of SMU-YAK-J1 was analyzed using all the BNoV genomes available in GenBank. The putative major parental strain (green) is Bo/HN-1/2018 (GenBank accession number MN122335.1), the yellow (unknown sequence) is the minor parental strain, the purple is Newbury2/1976/UK (GenBank accession number AF097917), and 4962 nt is the predicted recombination site.

A nearly full-length genome containing 7272 nt and three complete ORFs was successfully obtained from a BNoV-positive sample; this BNoV was named SMU-YAK-J1. The G + C content of the SMU-YAK-J1 genome was 57.7%. ORF1 was 5050 nt in length and encoded 1684 amino acids, which form nonstructural proteins. ORF2 was 1574 nt in length and encoded 524 amino acids, which form the VP1 protein. ORF3 was 639 nt in length and encoded 212 amino acids, which form the VP2 protein (Figure 1A).

SMU-YAK-J1 shares 90.9% nt identity with the only genome of GIII.P2_GIII.4, shares 79.5–85.9% nt identity with seven complete genomes of GIII.P2_GIII.2 and shares 71.6% nt identity with the sole complete genome of GIII.P1_GIII.1 in the GenBank database. Although the genome of SMU-YAK-J1 is most genetically related with the sole GIII.P2_GIII.4 genome, there are also many unique aa distinguishments (Figure 1B). Aa identities of the nonstructural proteins and structural proteins of SMU-YAK-J1 with those of nine other GIIIs are summarized in Table 1, and the allelic differences of amino acids among the different genotypes are shown in Table S1. Taken together, SMU-YAK-J1 belongs to genotype GIII.P2_GIII.4.

### 2.3. Recombination Analysis of SMU-YAK-J1

To analyze the genetic recombination of SMU-YAK-J1, different sequences from the BNoV genome, complete ORF1/ORF2 region and complete ORF2/ORF3 region were used by Recombination Detection Program (RDP) 4.0. The results showed that the recombination event occurred only when using complete ORF1/ORF2 sequences, which was supported by RDP, Chimaera, BootScan, 3Seq, GeneConv, MaxChi and SiScan, with a recombinant score of 0.587. A recombination breakpoint was predicted at nt 4962 in the genome which was located at the 3′ end of ORF1 and the 5′-end of ORF2. The putative major parental strain was Bo/HN-1/2018 (GenBank accession number MN122335.1), but the minor parental strain was not found (Figure 1C).

### 2.4. Characterization of RdRp of SMU-YAK-J1

**Figure 2:**
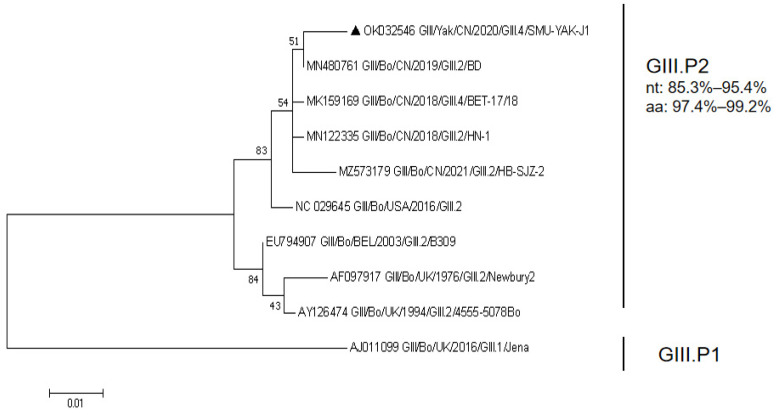
Phylogenetic tree constructed with all the full-length RdRp sequences available in GenBank and the full-length RdRp sequence of this study. Sequence alignments and clustering were performed using ClustalW in MEGA 7.0. The tree was constructed using the maximum likelihood method with bootstrap values calculated for 1000 replicates. The BNoVs in this study were marked with triangles in the phylogenetic tree. Nt represents nucleotide identity in the same genotype, and aa represents amino acid identity in the same genotype.

RdRp of SMU-YAK-J1 is 1518 bp in length, encodes 506 amino acids. It shares 85.3–97.0% nt identity and 89.7–99.6% aa identity with the GIII.P2, and 74.7% nt identity and 89.1% aa identity with the GIII.P1 in GenBank. A maximum likelihood phylogenetic tree constructed with all the full-length RdRp sequences available in GenBank and the full-length RdRp sequence of this study indicated that SMU-YAK-J1 and four Chinese BNoVs clustered into one large branch and SMU-YAK-J1 and Bo/CH/HB/BD/2019 (GIII.P2_GIII.2) clustered into an independent small branch (Figure 2). 

### 2.5. Characterization of the VP1 Protein of SMU-YAK-J1 

**Figure 3:**
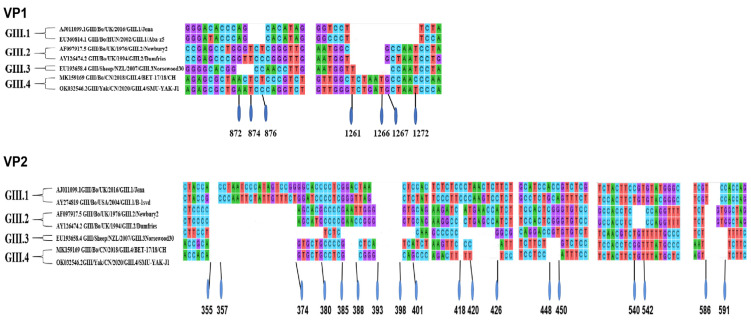
Insertion/deletion sites in VP1 and VP2 between the GIII.4 sequence and other genotypes. The BNoVs in this study were marked with triangles in the figure.

The complete VP1 protein of SMU-YAK-J1 is 1575 nt in length and encodes 525 amino acids (as long as that in case of GIII.4). Interestingly, sequence insertions were found in GIII.4 compared with other genotypes. The complete VP1 of SMU-YAK-J1 and Bo/BET–17/18/CH are 15 nt longer than that of GIII.1, 6 nt longer than that of GIII.2 and 9 nt longer than that of GIII.3. The specific insertion sites are shown in Figure 3. SMU-YAK-J1 shared 82.7–83.2% nt identity and 93.9–94.3% aa identity with the potential GIII.4, 67.1–68.9% nt identity and 70.8–72.9% aa identity with the GIII.1, 66.3–69.0% nt identity and 69.8–72.9% aa identity with the GIII.2, 69.0–70.0% nt identity and 71.4–72.1% aa identity with the only GIII.3 in GenBank. 

**Figure 4:**
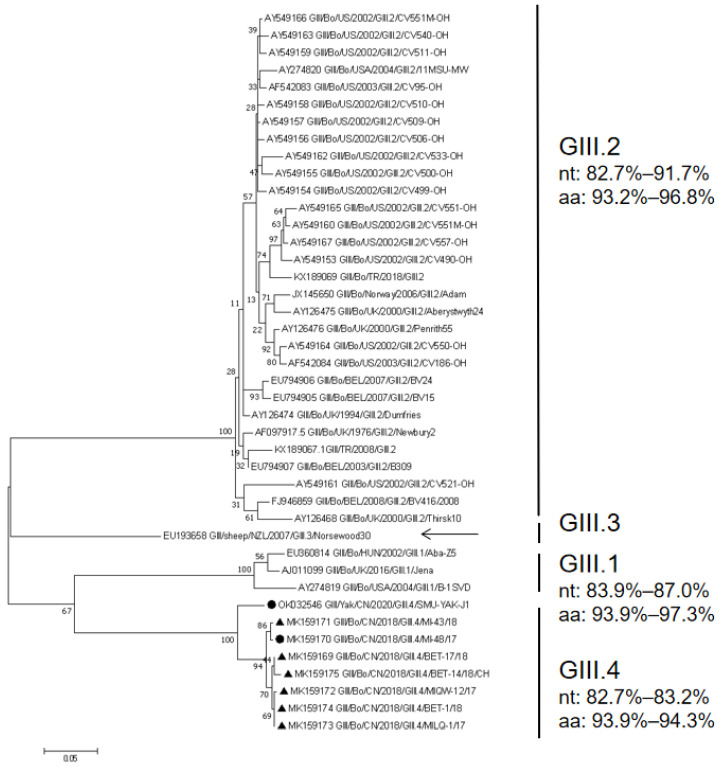
Phylogenetic tree based on all the complete VP1 amino acid sequences available in GenBank and the counterpart of this study. Sequence alignments and clustering were performed using ClustalW in MEGA 7.0. The tree was constructed using the maximum likelihood method with bootstrap values calculated for 1000 replicates. The BNoVs in this study were marked with circles, and seven BNoVs from GIII.4 were marked with triangles in the phylogenetic tree of VP1. Nt represents nucleotide identity in the same genotype, and aa represents amino acids identity in the same genotype.

A maximum likelihood phylogenetic tree constructed on the basis of the complete VP1 sequences of SMU-YAK-J1 and all the complete VP1 sequences available in GenBank showed that SMU-YAK-J1 and the other seven GIII.4 clustered independently into one large clade, but independently into a small clade, which is less genetically distant from other GIII.1 genomes and very distant from GIII.2 and GIII.3 (Figure 4). SMU-YAK-J1, combined with seven GIII.4, formed an independent large branch, but SMU-YAK-J1 was independently clustered into a small branch.

Moreover, GIII.P2_GIII.4 and the other genotypes shared 44 identical aa mutations among the 525 aa of the VP1 protein shown in Table S1, SMU-YAK-J1 and the seven GIII.4 genomes shared 26 identical aa mutations among the 525 aa of the VP1 proteins shown in Figure 1B. 

### 2.6. Characterization of the VP2 Protein of SMU-YAK-J1

The VP2 protein of SMU-YAK-J1 is 639 nt in length and encodes 212 amino acid sequences (as long as that in case of GIII.P2_GIII.4). The VP2 proteins of SMU-YAK-J1 and Bo/BET–17/18/CH were 33 nt shorter than those of GIII.P1_GIII.1 and 12 nt shorter than those of the GIII.P2_GIII.2 shown in Figure 3. SMU-YAK-J1 shared 82.2% nucleotides (nt) identity (97.1% aa identity) with the GIII.P2_GIII.4, 64.8–66.9% nt identity (81.7–86.5% aa identity) with the GIII.P2_GIII.2 in the GenBank database, 58.5–59.1% nt identity (81.7% aa identity) with the GIII.P1_GIII.1, 63.4% nt identity (72.1% aa identity) with the GIII.3 which is identified in sheep feces. 

**Figure 5:**
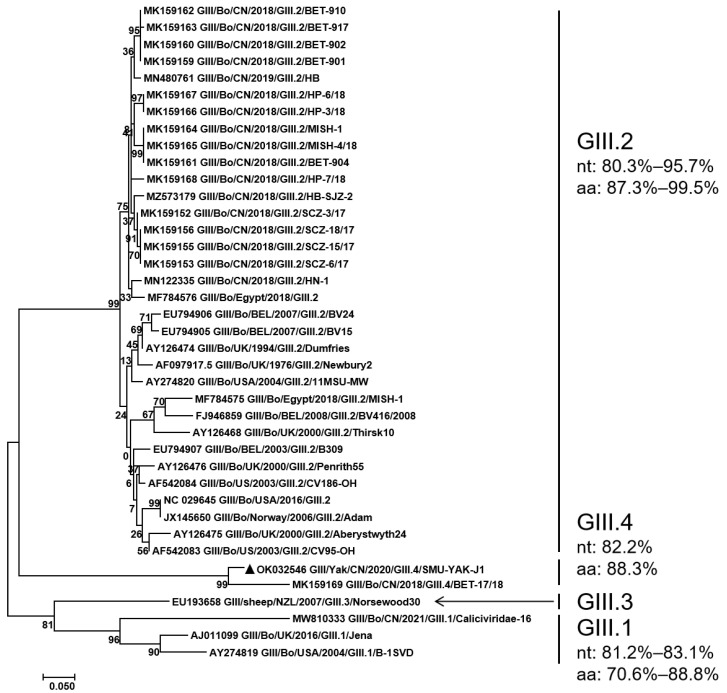
Phylogenetic tree based on the ≥ 639 bp VP2 amino acid sequences. Sequence alignments and clustering were performed using ClustalW in MEGA 7.0. The tree was constructed using the maximum likelihood method with bootstrap values calculated for 1000 replicates. The BNoVs in this study were marked with triangles. Nt represents nucleotide identity in the same genotype, and aa represents amino acid identity in the same genotype.

In a maximum likelihood phylogenetic tree constructed with all the sequences of the BNoV VP2 gene that were ≥ 639 bp available in GenBank and the counterpart of this study, SMU-YAK-J1 clustered on a unique branch with GIII.4 (Figure 5), which is genetically distant from other genotypes. GIII.P2_GIII.4 and the other genotypes shared 30 identical aa mutations among the 212 aa of the VP2 protein shown in Table S1. SMU-YAK-J1 and GIII.4 shared 20 identical aa mutations among the 212 aa of the VP2 protein shown in Figure 1B. 

## 3. Discussion

### 3.1. First Identification of BNoV in Yak

BNoV has emerged as a viral pathogen that causes a gastrointestinal illness and diarrhea in cattle, either alone or with other viral enteropathogens worldwide [1,11,34]. Yak are essential animals that support the livelihood of the local Tibetan people, and diarrhea is a common disease in yak [28,29,30,31,32,33]. However, there is no information regarding NoV infection in yak. In this study, to understand the existence and prevalence of BNoV in yak, BNoV detection was conducted in 205 diarrhea samples, and four (1.95%) were detected as pathogen-positive, distributed at four out of the ten farms; the farthest geographical distance between the two BNoV-positive farms was > 300 km, indicating that BNoV, despite not being the major causative agent of diarrhea, has been circulating among yak with wide geographical distribution in China. This study first determined the presence of BNoV in yak, and further surveillance is needed to monitor its prevalence.

### 3.2. Genome Characteristics of BNoV in Yak

A nearly full-length genome of SMU-YAK-J1-20 was successfully sequenced. Sequence analysis with only nine genome sequences of the GIII genogroup showed that SMU-YAK-J1 was most related with GIII.P2 GIII.4, sharing 90.9% genomic nucleotide identity, but only shared 71.6–85.9% identity with the others, which confirmed that SMU-YAK-J1 belongs to genotype GIII.P2_GIII.4. However, compared with the sole genome of GIII.4 in GenBank, the BNoV in this study also exhibited many unique amino acid changes among all the three ORFs. Besides, natural genetic recombination and sequence insertion and deletion in the genome of SMU-YAK-J1 were also observed. All these molecular characteristics may contribute to a better understanding of the prevalence and genetic evolution of BNoV.

### 3.3. Recombination Event of BNoV in Yak

Previous studies have shown that human NoVs were identified in bovine stool specimens [35]; besides, calves were also susceptible to the same genotype, but genetically different [36]. All this evidence indicated that superinfection and coinfection with multiple genogroups and genotypes may occur, facilitating natural genetic recombination of BNoVs, and recombination may be a common mechanism for the generation of a diversity of genotypes or genogroups [37]. In this study, a recombination event was observed in SMU-YAK-J1. The major parent was Bo/HN-1/2018 (GIII.P2_GIII.2), while the minor parent was not found (Figure 1C). The phenomena have been reported in many NoVs from humans, bovines and pigs [37,38,39], which may be caused by limited NoVs. Herein, gene conversion occurred between ORF1 and ORF2, which is consistent with the findings that the putative region of recombination in human NoVs and BNoV was suggested to be the ORF1/ORF2 junction region [40,41,42]. Recombination at this junction allows the virus to alter its viral capsid while retaining the region involved in the replication of the viral genome. As SMU-YAK-J1 has the highest homology with the only GIII.4 in the ORF2 domain, SMU-YAK-J1 likely was a recombinant generated by Bo/BET–17/18/CH (GIII.P2_GIII.2) for ORF1 and by Bo/BET-17/18/CH (GIII.P2_GIII.4) for ORF2. The reason for not recognizing Bo/BET-17/18/CH (GIII.P2_GIII.4) as the minor parent strain may be induced by the further aa mutations (Figure 1B) of SMU-YAK-J1 in ORF2, which is the domain for host specificity [40], to better adapt to the unique yak in the Tibetan Plateau.

### 3.4. Sequence Characteristics in the VP1 and VP2 Domains of SMU-YAK-J1

Another interesting finding is that there are unique sequence insertions and deletions in the VP1 and VP2 genes of GIII.4 compared with other genotypes. The capsid protein VP1 of NoVs is composed of two structural domains, the S (1–225 aa; the most conserved region for genotyping NoVs and discovering a recombinant [36,43]) and P domains (226–525 aa; the least conserved region that is involved in receptor binding and infective ability of NoVs). The P domain is further divided into P1A (226–278 aa), P2 (279–405 aa) and P1B (406–525 aa) [36]. The sequence insertion sites of genotype GIII.4 were located in P2 and P1B, respectively. A previous study showed that the P2 subdomain of bovine GIII.2 was relatively similar to human G1.1, both having deletions in the P2 subdomain and showing a relatively smaller interface surface [36,44]. Amino acid sequence alignment indicated that GIII.4 has the same P2 length as GIII.2, one aa longer than both GIII.1 and GIII.3, implying that genotype GIII.4 may have a similar P2 function to GIII.2 and even human GI.1 [44,45]. In the P1B subdomain, the insertion sequences associated with the binding of HBGAs [21,46] were located in the interface loop and were 4 aa longer than GIII.1 and one aa in different positions longer than GIII.2 and GIII.3, respectively. This increasingly longer aa sequence dramatically contributes to a longer interface loop than GIII.1, GIII.2 and GIII.3 to different degrees, which has also been confirmed in the only GIII.4 genome from a dairy cow [8]. These sequence insertions in the P domain may affect the ability of receptor binding with the host and some other functions. Further studies are needed in order to determine the role of sequence insertions on the P domain and even NoVs.

Different sequence insertions and deletions were also observed in VP2 compared with GIII.1, GIII.2 and GIII.3. While the precise role of VP2 remains to be elucidated, there is evidence that it might be involved in particle stabilization [47,48]. Therefore, whether these sequence variations could affect the stability of NoV particles also needs to be further investigated. It is doubtless that each genotype has unique molecular features of sequence insertion and deletion, presenting a coevolutionary trend in VP1 and VP2. Nevertheless, there are still many aa differences in the same genotype, just as the sequence alignment result of two GIII.4. These aa variants of SMU-YAK-J1 may represent the adaption mutation in yak in the Tibetan Plateau. 

## 4. Conclusions

In this study, four of the 205 diarrhea samples from four of the ten farms tested positive for BNoV; this is the first time BNoV infection was reported in yak. To gain insights into the molecular characteristics of SMU-YAK-J1, its genome was successfully obtained and analyzed. The genome was most related with GIII.P2_GIII.4, which confirmed that SMU-YAK-J1 belongs to GIII.P2_GIII.4. However, compared with the sole genome of GIII.4 in GenBank, the BNoVs in this study all exhibited many unique amino acid changes among all the three ORFs, which may represent the unique genetic evolution in yak. This study first determined the presence of BNoV in yak, contributing to a better understanding of the prevalence and genetic evolution of BNoV.

## 5. Materials and Methods

### 5.1. Specimen Collection

From May 2018 to October 2020, a total of 205 yak diarrhea samples (aged 3 months) were collected from 10 farms in Sichuan Province (altitudes ranging from 3500 to 4500 m). The farms were located in Hongyuan County (N 32°47′, E 102°32′) and Norgay County (N 34°10′, E 102°32′). All of the samples were kept on ice at a temperature of −80 °C.

### 5.2. RNA Extraction and cDNA Synthesis

The clinical fecal samples were thoroughly resuspended in PBS (1:5) and centrifuged at 10,000× *g* for 10 min before filtering through a 0.45 m filter. RNAios Plus (TaKaRa Bio Inc., Kusatsu, Japan) was used to extract viral RNA from 300 l of the fecal solution according to the manufacturer’s instructions. According to the manufacturer’s instructions, cDNA was generated using a PrimeScriptTM RT Reagent kit (TaKaRa Bio Inc.); 4 l 5Prime Script Buffer, 1 l Prime Script RT Enyme MixI, 2 l Random 6 mers, 9 l RNase Free H_2_O and 4 l RNA were used in the reaction volume for cDNA synthesis. The mixes were then baked at 37 °C for 15 min, 85 °C for 15 s and 16 °C for 10 min before being stored at 20 °C.

### 5.3. Detection of BNoV

According to a prior report [34], BNoV was discovered using a particular RT-PCR assay targeting RdRp. The primer sequences were as follows:

CBECU-F: 5′-AGTTAYTTTTCCTTYTAYGGBGA-3′

CBECU-R: 5′-AGTGTCTCTGTCAGTCATCTTCAT-3′, and the amplified fragment was 532 bp. The amplification products were examined using 1.5% agarose gel electrophoresis, and all the BNoV-positive products were sequenced directly in both directions by Tsingke Biotechnology Co., Ltd. (Chengdu, China), to ensure that the detection findings were accurate.

### 5.4. Genome Amplification of BNoV

According to the BNoV sequence available in GenBank, 12 pairs of primers were designed to successfully amplify SMU-YAK-J1 (GenBank accession number OK032546). An Omega Gel kit (Omega, New York, NY, USA) was used to purify all the PCR products, which were then cloned into the pMD19-T simple vector (TaKaRa Bio Inc.) and transformed into DH5 competent *Escherichia coli* cells (Yeasen, Shanghai, China). Three to five colonies were chosen for each product and sequenced in both directions (Sangon, Shanghai, China). Another nine pairs of primers targeting various locations were constructed to amplify the given genomic sequence in order to validate it. Tables S2 and S3 provide the sequence information for the PCR primers used for genome amplification.

### 5.5. Sequence, Phylogeny and Recombination Analysis

SeqMan was used to put together the sequences (version 7.0; DNASTAR Inc., Madison, WI, USA). ORF Finder (http://www.ncbi.nlm.nih.gov/gorf/gorf.html, accessed on 30 August 2021) was used to determine the potential ORFs and their associated amino acids for the genome organization study. The MegAlign tool of DNASTAR 7.0 was used to identify homologies of nucleotides and deduced amino acid sequences (DNASTAR Inc.). MEGA 7.0 was used to generate multiple nucleotide alignments and create a maximum likelihood phylogenetic tree with bootstrap support. Recombination Detection Program 4.0 (RDP 4.0, version 4.96) was utilized for recombination analysis, which included the RDP, GeneConv, Chimaera, MaxChi, BootScan, SiScan, and 3Seq methods [49].

## Figures and Tables

**Table 1:** Amino acid identities of SMU/YAK-J1 compared with nine GIII genomes.

BNoV	Genotype	GenBank Accession Number	Genome Length (nt)	Genome Nucleotide Identities	Amino Acid Identities (%)								
					ORF1						ORF2		ORF3
					P48	NTPase	P22	VPg	Pro	RdRp	S	P	VP2
NC029645	GIII.P2_GIII.2	NC029645.1	7313	80.6	92.4	98.1	94.6	95.2	97.8	98.6	83.3	57.9	65.9
Bo/CH/HB/BD/2019	GIII.P2_GIII.2	MN480761.1	7320	85.4	98.2	99.4	93.5	97.6	100	99.2	88.9	56.5	66.8
Bo/HN-1/2018	GIII.P2_GIII.2	MN122335.1	7342	86.1	97.0	98.6	96.7	96.8	98.9	99.0	86.3	57.9	66.8
Bo/GIII.2/Adam/2006	GIII.P2_GIII.2	JX145650.1	7313	80.6	92.4	98.1	94.6	95.2	97.8	98.6	83.3	57.9	65.9
Bo/GIII/B309/2003/BEL	GIII.P2_GIII.2	EU794907.1	7317	79.2	88.8	98.1	95.7	93.7	98.3	97.8	82.9	59.2	67.3
Bo/Dumfries/94/UK	GIII.P2_GIII.2	AY126474.2	7311	79.2	90.3	98.6	95.1	93.7	98.3	97.4	82.5	58.2	66.8
Jena	GIII.P1_GIII.1	AJ011099.1	7338	70.5	69.8	87.9	69.6	77.8	87.8	89.1	87.7	60.2	62.8
Bo/Newbury2/1976/UK	GIII.P2_GIII.2	AF097917.5	7311	79.1	90.3	98.3	95.1	94.4	98.3	97.4	82.1	57.8	67.3
Bo/BET-17/18/CH	GIII.P2_GIII.4	MK159169.1	7321	91.0	96.7	98.6	97.8	97.6	99.4	98.8	97.6	91.3	90.1

## Data Availability

All the sequencing results in this study were submitted to the GenBank database under accession numbers OK032546, OL831112 and MN688697-MN688698.

## Supplementary Materials

The following are available online at www.mdpi.com/article/10.3390/pathogens11020192/s1, Supplementary Table S1: The identical aa mutations of GIII.P2_GIII.4 and the other genotypes; Supplementary Table S2: Primer sequences used for genomic amplification and sequencing; Supplementary Table S3: Primer sequences used to verify the complete genome sequences.
