# Supplementary material for: First Detection and Genomic Characterization of Bovine Norovirus from Yak
Source: Pathogens. 2022 Jan 31;11(2):192. doi: 10.3390/pathogens11020192 (PMC8874446; doi:10.3390/pathogens11020192)
Supplement: Supplementary file 1 [file pathogens-11-00192-s001.zip › pathogens-1533938-supplementary.pdf]

Table S1. The identical aa mutations of GIII.P2_GIII.4 strain and the other genotypes strains .

Genotype	Amino Acid Difference Site in VP1	Amino Acid Difference Site in VP2
GIII.1	L79M,V121L,A150T,A159V,S233A,V241F, V244S,Q245D,L252H,Q257R,D268E,P274P,A275P, S276C,F283M,V284L,A288E,E289S,T290A,A295F, A304V,L314E,S323R,V328F,A329YV334Y,W335Y, Q336T,S338D,K342S,N346R,S357E,G358S,M360L, R364Q,D370S,Y371H,P375A,T376G,D378L,S379A, I383P,S388G,S393A,V404I,C413S,N414S,L418E, L432M,F440L,S473P,L476I,L488F,T490L,K495R	T3A,D28E,S39Q,Q46A,D65A,F66Y,A70S,V72M, Q73A,F76Y,A78E,V85S,V88G,I94L,V108Q,L117T, A119C,S137T,S141N,,146A,T179G,S180L,A182Y, S183T,Y184S,S186F,S187N,F188Q,N189G,L190W, G191F,W192N,N194D,T195R,D196M,R197P
GIII.2	L79M,V121L,A150T,E159V,N233A,E241F,A244S, Q245D,F252H,Q257R,D268E,P274T,S275A,S276C, F283M,V284L,E289S,P290A,V295F,D304V,F314E,S323R, V328F,A329Y,V334Y,G335Y,E335Y,E336T,T338D,R342S, N346R,D357E,E358S,A360L,L364Q,R370S,N371H, P375A,T376G,H378L,S379A,I383P,A388G,S393A, V404I,C413S,N414S,L418E,L432M,F440L,N473P,L476I, L488F,V490L,K495R	S3A,D28E,A39Q,E46A,D65A,F66Y,A70S,Q72M,E73A, G78E,V85S,V88G,V94L,V108Q,L117T,D119C,S137T, R141N,H146A,A179G,S180L,A182Y,S183T,Y184S,S186S, S186F,S187N,F188Q,N189G,L190W,G191F,W192N,N194 D, T195R,D196M,R197P
GIII.3	L49M,V121L,A150T,E159V,S233A,E241,A244S,Q245D, F252H,Q257R,D268E,P274T,S275P,S276C,F283M,V284L, G288E,G289S,T290A,A295F,A304V,F314E,T323R,L328F, A329Y,L334Y,E335Y,H336T,H338D,T342S,N346R,D357E, G358S,M360L,L364Q,E370S,G371H,P375A,S376G,N378L, T39A,M383P,A388G,S393A,V404I,C413S,N414S,H418E, L432M,L404F,A473P.L476I,L488F,V490L,K495R	T3A,D28E,S39Q,S46A,E65A,F66Y,A70S,I72M,S73A,F76Y, Q78E,V85S,A88G,V94L,V108Q,L117T,A119C,S137T,R141 N, M146A,F179G,S180L,A182Y,S183T,Y184S,S186F,S187N, F188Q,N189G,L190W,G191F,W192N,F193T,N194D,T195R , D196M,R197P

Note:There is no identical aa mutations between GIII.P2_GIII.4 and the other genotypes strains.

Table S2. Primers sequences used for genomic amplification and sequencing .

Primer Name	Nucleotide sequence(5′→3′)	Amplicon (bp)	Location[a]	Annealing Temperature (℃)
J1-F	GTGAATGAAGACTTTGACGATATGG	1227	1-1227	55
J1-R	CTCGACGCGCTTCAGGGTCTGGCC			
J2-F	AGATATTCAACATAATCAAGA	493	1160-1672	55
J2-R	AGTCTGCGGCAATGTTGGCCATG			
J3-F	GCCCCCTGGCATCGGGAAGACC	1458	1491-2948	56
J3-R	GGGTTCCAGTCAATCTTTCTCC			
J4-F	ATCAGAGAGGAGCGGGGTGGCAACTA	761	2764-3524	63
J4-R	ATGGCACAGATGACTGTGTTGCCAGA			
J5-F	GCAACACAGTCATCTGTGCC	411	3503-3913	57
J5-R	GATAGCCAGAGCTGGTGGTC			
J6-F	GCCTCGAACAGGTGATGGACCA	1429	3827-5256	62
J6-R	GTAAACTCMCCCTGGGGGGC			
J7-F	TCCCGATTTTGTAAATGAAGATGACT	419	5049-5467	54
J7-R	TGGGGTGAGAAAGGAGGTATCA			
J8-F	ATGCGCGTTAGAGTGATC	817	5369-6185	53
J8-R	CCTGCTGAACATGAGAAAGGA			
J9-F	CGCATGTAYAACGGCTGGAC	908	5342-6249	58
J9-R	GCAAACTCTGCTCCAAGAACGT			
J10-F	TGGGAACCCCCAGACTATCGTG	425	6203-6627	58
J10-R	TTAATCCGAGGAAGACGACGG			
J11-F	TGGGAAATGTTAGTGTTGGCCGCC	400	6588-6988	63
J11-R	GGCAGCACTGTGGTGAAGCCACCG			
J12-F	GCTGCTTGATTGGAATGG	420	6919-- 7321	50
J12-R	TAACAGCAAGAATAGGGGAAAA			

Table S3. Primers sequences used for verify the complete genome sequences.

Primer Name	Nucleotide sequence(5′→3′)	Amplicon (bp)	Location[a]	Annealing Temperature (℃)
J1-1-F	GTGAATGAAGACTTTGACGA	1064	1-1064	53
J1-1-R	AGCCCAACTCCACCAAGTAT			
J2-1-F	AGATGTGGCCAACTTCTTGG	920	960-1879	56
J2-1-R	GATTCTCGATTTCCTGAGAGTTG			
J3-1-F	CTGGACCACATGAACCTGGG	980	1801-2780	58
J3-1-R	CCCCGCTCCTCTCTGATTTT			
J4-1-F	CGGAAGACCAATGCCTTCT	1040	2701-3740	57
J4-1-R	CGCATGACCTGTTGTAGGGA			
J5-1-F	CACCTATGAGCCAGCCTATC	993	3666-4658	56
J5-1-R	GTCGGCTTGAGGCCATACC			
J6-1-F	TCTTGAGTTTGATCCAGACAAG	972	4599-5570	55
J6-1-R	GCCCCTGGTGAAAAAGGATG			
J7-1-F	CATTGTTGACGTCCGCAC	1043	5494-6536	56
J7-1-R	CAGAAAGGGGGAAGGTCACC			
J8-1-F	CTTACTGAACTATGTGAGCCC	333	6418-6750	
J8-1-R	AGGAGCGCTGTTGGAGTT			56
J9-1-F	TAATGGCTGCTGAGTTCTTT	666	6625-7290	
J9-1-R	GAAATAAAACTCACTGCTCACT			53
